# Purkinje cell responses during visually and vestibularly driven smooth eye movements in mice

**DOI:** 10.1002/brb3.310

**Published:** 2015-01-21

**Authors:** Akira Katoh, Soon-Lim Shin, Rhea R Kimpo, Jacob M Rinaldi, Jennifer L Raymond

**Affiliations:** Department of Neurobiology, Stanford University299 W. Campus Drive, Stanford, California, 94305-5125

**Keywords:** Cerebellum, extracellular recording, oculomotor, optokinetic reflex, vestibulo-ocular reflex

## Abstract

**Introduction:**

An essential complement to molecular-genetic approaches for analyzing the function of the oculomotor circuitry in mice is an understanding of sensory and motor signal processing in the circuit. Although there has been extensive analysis of the signals carried by neurons in the oculomotor circuits of species, such as monkeys, rabbits and goldfish, relatively little in vivo physiology has been done in the oculomotor circuitry of mice. We analyzed the contribution of vestibular and nonvestibular signals to the responses of individual Purkinje cells in the cerebellar flocculus of mice.

**Methods:**

We recorded Purkinje cells in the cerebellar flocculus of C57BL/6 mice during eye movement responses to vestibular and visual stimulation.

**Results:**

As in other species, most individual Purkinje cells in mice carried both vestibular and nonvestibular signals, and the most common response across cells was an increase in firing in response to ipsiversive eye movement or ipsiversive head movement. When both the head and eyes were moving, the Purkinje cell responses were approximated as a linear summation of head and eye velocity inputs. Unlike other species, floccular Purkinje cells in mice were considerably more sensitive to eye velocity than head velocity.

**Conclusions:**

The signal content of Purkinje cells in the cerebellar flocculus of mice was qualitatively similar to that in other species. However, the eye velocity sensitivity was higher than in other species, which may reflect a tuning to the smaller range of eye velocities in mice.

## Introduction

The cerebellum contains roughly half of all neurons in the brain (Lange 1975), yet it has a relatively simple circuit architecture, which makes it a tractable model for studying the function of neural circuits. The cerebellum has both cognitive and motor functions (Ivry and Baldo 1992; Thach 2007; Ito 2008; Schmahmann 2010). Its motor functions are frequently studied using oculomotor behavioral tasks, such as the vestibulo-ocular reflex (VOR). The VOR is a reflex which stabilizes images on the retina by generating an eye movement in the direction opposite from head movement. Studies of the VOR in a variety of species, including monkeys, rabbits, cats, and goldfish, have yielded numerous insights about sensory and motor signal processing in the cerebellar circuit during the induction and expression of motor learning (du Lac et al. 1995). With the explosion of molecular-genetic approaches for analyzing neural circuits, the VOR is being studied in mice as well. Mice exhibit robust VOR learning, comparable to that reported previously in other species (Koekkoek et al. 1997; Katoh et al. 1998; Iwashita et al. 2001; Boyden and Raymond 2003). A critical complement to the use of molecular-genetic approaches in mice is an understanding of the signal processing in the VOR circuit.

In other species, the signal content of cerebellar neurons during the VOR has been extensively analyzed. Both vestibular and nonvestibular signals are encoded by neurons in the relevant part of the cerebellum, the flocculus (Lisberger and Fuchs 1974; Robinson 1976; Collewijn and Grootendorst 1979; Ito et al. 1979; Miles and Eighmy 1980; Godaux et al. 1983; Nagao 1983; Noda 1986; Pastor et al. 1997; Raymond and Lisberger 1997; Blazquez et al. 2003; Arenz et al. 2008; Ke et al. 2009). The nonvestibular signals are correlated with eye velocity, and could reflect an efference copy of the eye movement command, arising from the medial vestibular nucleus and nucleus prepositus hypoglossi (Lisberger and Fuchs 1978b; Miles and Braitman 1980; McCrea et al. 1987ab[Bibr b43],[Bibr b44]; Buttner-Ennever et al. 1989; Hirata and Highstein 2001; Kolkman et al. 2011), visual signals encoding image motion on the retina (Maekawa and Takeda 1975; Miyashita et al. 1980; Noda 1981; Waespe and Henn 1981; Blanks and Precht 1983; Kawano et al. 1992; Frens et al. 2001) or both (Graf et al. 1988; Hirata and Highstein 2001). Individual Purkinje cells, which are the output neurons of the cerebellar cortex, can carry both vestibular and nonvestibular signals, and their responses during the VOR seem to reflect a linear combination of the two signals (Lisberger and Fuchs 1978a; Miles et al. 1980; Stone and Lisberger 1990b; Pastor et al. 1997; Hirata and Highstein 2000). Thus, the signal content of these neurons cannot be fully understood without comparing the responses across appropriate visual and vestibular test stimuli in awake behaving animals.

Here, we apply methods that have been used in other species to assess the contribution of vestibular and nonvestibular signals to the responses of individual Purkinje cells in mice during the VOR. Previous recordings from Purkinje cells in the flocculus of mice have demonstrated that these cells respond during vestibular stimulation (Grusser-Cornehls et al. 1995a,b), consistent with the known input to the flocculus from the vestibular nuclei as well as primary vestibular afferents (Lisberger and Fuchs 1974; Maekawa and Takeda 1975; Waespe and Henn 1981; Blanks et al. 1983; Grusser-Cornehls et al. 1995b; Arenz et al. 2008). However, these previous recordings were made in paralyzed mice that were not actually performing the VOR behavior. More recently, several studies have recorded from floccular Purkinje cells of awake behaving mice, during visually driven eye movements (Goossens et al. 2004; Hoebeek et al. 2005; Yoshida et al. 2007) and during the VOR (Clopath et al. 2014; Stahl and Thumser 2014). Here, we extend these previous studies by analyzing the signal content of the simple spike responses of flocculur Purkinje cells in mice, through a comparison of their responses during a set of paradigms designed to isolate the contribution of vestibular and nonvestibular signals to the responses of these neurons.

## Materials and Methods

### Animal preparation

Experiments were performed on 80 C57BL/6 adult mice (≥8 weeks old) from Charles River Laboratories (USA). Mice were first implanted with a head post and scleral search coil (Boyden and Raymond 2003). While the mouse was under anesthesia, a custom-built head post was attached to the top of the skull using anchor screws and dental acrylic, and a scleral search coil (IET, Marly, Switzerland) weighing ∼50 mg was implanted on the temporal side of the right eye beneath the conjunctiva. The search coil leads were run subcutaneously to a two-pin connector. After several days of recovery, a 1 mm diameter craniotomy was made, under anesthesia, on the left periotic capsule, which overlies the floccular complex, using an approach through the pinna. A Teflon cannula was placed into the hole at an angle parallel to the earth and 25° posterior to the interaural axis, and affixed with dental acrylic. The cannula did not obstruct the visual field of the mouse. Mice were allowed to recover from surgery for 5–7 days before oculomotor testing. All animal protocols were approved by Stanford University's IACUC, the Administrative Panel for Laboratory Animal Care.

### Electrophysiology

**Figure 1:**
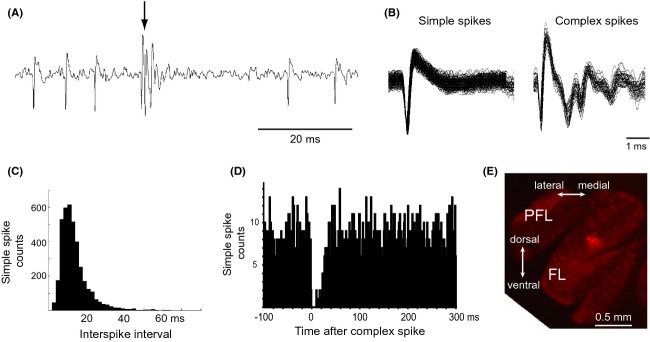
Extracellular single-unit recording from a representative Purkinje cell in the cerebellar flocculus of an awake mouse. All panels are from the same cell. (A) Recording from an isolated Purkinje cell showing a complex spike (arrow) and simple spikes. (B) Overlaid waveforms of isolated simple spikes and complex spikes. (C) Histogram of interspike intervals from the Purkinje cell. In this cell, mean firing rate was 74.5 sp/s and CV was 0.54. (D) A complex spike-triggered average of simple spike firing reveals the characteristic complex spike-triggered pause of simple spike activity. In this cell, mean pause was 31.5 ms. (E) Recording site, verified with injection of TexasRed conjugated with Dextran at the end of the recording session. FL: flocculus; PFL: paraflocculus.

Simultaneous recordings of eye movements and single unit activity were performed one to four times in a single mouse. Each recording session lasted from 10 to 60 min. Recording sessions in a single mouse were separated by at least 48 h, and we recorded at most four Purkinje cells from a single mouse. Extracellular single-unit activity was recorded using a borosilicate micropipette (0.7 mm-OD, FHC, Bowdoin, ME) filled with 2 mol/L NaCl (1–3 MΩ), which was inserted into the flocculus for each recording session through the implanted cannula. The signal was amplified, and band-pass filtered between 0.3 and 3 kHz (Dagan, Minneapolis, MI). Action potentials were detected with a hardware window discriminator (Bak Electronics, Mount Airy, MD), and the times of the resulting pulses were recorded to the nearest 10 *μ*s. In addition, the signal from the electrode was recorded at a sampling rate of 50 kHz for use in offline spike sorting. Purkinje cells were identified using previously established criteria (Miles et al. 1980; Goossens et al. 2004; Yoshida et al. 2004) (Fig.1A–D). The presence of complex spikes aided in the identification of Purkinje cells, however, the experimental conditions were optimized for analyzing the signal content of the Purkinje cell simple spike responses, hence the complex spike responses were not analyzed further. Purkinje cells were included in the analysis if their simple spike firing rate was significantly modulated by at least one of three test stimuli (see below).

The flocculus was targeted using stereotaxic coordinates and electrophysiological landmarks such as the characteristically high level of activity in the cerebellar cortex, and the number of layers of cerebellar cortex recorded during the electrode penetration. In 18 animals, the recording site was verified after recording via iontophoresis of 0.5% of Dextran-Texas Red (Molecular Probes, Eugene, OR) dissolved in 2 mol/L NaCl at the recording site, using the same electrode as for recording. For iontophoresis, 3–10 ms biphasic voltage pulses of 50–80 V were delivered at 100 Hz for 5 min. The mouse was anesthetized deeply and perfused with 0.1 mol/L phosphate buffer, pH 7.4, containing 4% paraformaldehyde. The cerebellum was embedded in optimal cutting temperature compound (Tissue-Tek, Torrance, CA), 50 *μ*m slices were prepared on a cryostat, and localization of the recording site in the flocculus was confirmed on a fluorescence microscope (Fig.1E). Of 18 brains sectioned after recording, 17 revealed Dextran-Texas Red in the flocculus, whereas one had no obvious fluorescent signal anywhere in the brain, suggesting a failed injection. To map recording sites within the flocculus, we divided the flocculus into twelve compartments, based on three divisions in the rostral-caudal dimensions, and two divisions each in the dorsal-ventral and medio-lateral dimensions.Figure S1 indicates the distribution of our injections across the 12 compartments.

### Behavioral paradigms

The head of the mouse was immobilized by attaching the implanted head post to a restrainer. The restrainer was attached to a turntable (Carco Electronics IGTS, Pittsburgh, PA), which delivered a vestibular stimulus by rotating the mouse about an earth-vertical axis. Visual motion stimuli were delivered by a rotating optokinetic hemisphere made of a white translucent plastic half-dome with black and white vertical stripes, each of which subtended 7.5° of visual angle. The optokinetic hemisphere was back-lit by two fiber optic lights (JH Technologies, San Jose, CA). The eye coil method (Robinson 1963; Judge et al. 1980; Koekkoek et al. 1997) was used to measure eye movements. A set of 18-inch magnetic coils (CNC Engineering, Seattle, WA), fixed to the turntable, provided the signals for measuring eye position using the mouse's scleral search coil. Eye velocity was calculated from eye position with an analog differentiator and filter (corner frequency 300 Hz; designed by S.G. Lisberger). Signals were digitized at 500 Hz.

After a Purkinje cell was isolated, its responses were measured using a set of three test conditions: during the VOR in total darkness (VORD), during the optokinetic reflex (OKR), and as the mouse cancelled its VOR by tracking the optokinetic stimulus, which moved exactly with the head (VORC) (Fig. S2). The order of test stimuli was randomized for each mouse. For VORD and VORC the turntable was rotated about an earth-vertical axis, with a sinusoidal angular velocity of frequency 1 Hz, and peak speed of 10°/s. For VORC an illuminated optokinetic stimulus was rotated together with the turntable. To elicit the OKR, the optokinetic stimulus was rotated sinusoidally at 1 Hz, ±10°/s, with the head stationary. In addition, a subset of the cells were recorded in the absence of any vestibular or visual stimuli, while the turntable was stationary in the dark for a period of ∼60 s.

### Data analysis

Voltages related to the velocity of eye, head and optokinetic stimulus were recorded at 500 Hz per channel. Eye velocity recordings were edited to remove the rapid deflections caused by saccades and other movement artifacts. The data were then analyzed by aligning stimulus cycles on the velocity of the head (VORD and VORC) or visual stimulus (OKR) and averaging. All averages contained 10 cycles or more***.*** Values given in the text are mean ± SEM. Average eye and head velocity traces were subjected to Fourier analysis. The VOR gain was calculated as the ratio of eye velocity to head velocity at the fundamental frequency, and the VOR phase was calculated as the difference between the eye-velocity phase and the head-velocity phase in the opposite direction, with a perfectly compensatory VOR having a phase of zero. The OKR gain was calculated as the ratio of eye velocity to optokinetic stimulus velocity, and the OKR phase was calculated as the difference between the phase of the eye-velocity and the optokinetic stimulus velocity, with a perfectly compensatory OKR having a phase of zero. Spike frequency histograms (bin width: 2 ms) were subjected to Fourier analysis to calculate the amplitude and phase of Purkinje cells' responses at the fundamental frequency. Vector analysis was used to determine whether the firing rate modulation in a given Purkinje cell was significant. For this, the stimulus cycle was divided into 500 bins, with a vector assigned to each bin (each phase of the stimulus cycle) of length equal to the average firing rate in that bin. A Rayleigh's test was used to determine significance. Vector analysis was also used to calculate the mean and SEM of the responses across the population of Purkinje cells.

To calculate the sensitivity of each cell to eye velocity (*β*), the amplitude of the firing rate response during OKR was divided by the amplitude of the eye velocity response at the fundamental frequency. The sensitivity of each cell to head velocity (*α*) and the phase of the head velocity sensitivity (*θ*), were calculated using the following equation:


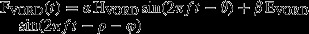
(1)

where F_VORD_ (t) is the fundamental component of the firing rate response during VORD, determined from the Fourier analysis; H_VORD_ and E_VORD_ are the peak head and eye velocity during VORD, also determined from the Fourier analysis; f is the stimulus frequency, 1 Hz; *ρ* is the phase of eye velocity sensitivity (equal to the phase of firing during the OKR); *φ* is the phase of eye velocity relative to head velocity we measured during VORD.

Linearity of the vestibular and nonvestibular (eye velocity) input signals was assessed by comparing the measured response of a Purkinje cell during VORC with the predicted response F_VORC_ (t), calculated as follows


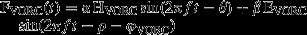
(2)

where *α* and *θ* were obtained from eqn. 2008, *β* and *ρ* from the Purkinje cell's response during OKR, and H_VORC_, E_VORC_ and *φ*_VORC_ are the peak head velocity, peak eye velocity, and phase of eye velocity relative to head velocity measured during VORC respectively.

Sensitivity to eye position (*γ*) was calculated from the correlation between the eye position and firing rate as animals made spontaneous, saccadic eye movements in the absence of vestibular and visual stimuli. A linear regression was performed on the eye position and firing rate, measured in 150-ms bins, as the animal sat stationary in the dark for ∼60 s. If the regression coefficient was significant (p < 0.05), the Purkinje cell was considered to be sensitive to eye position, and the regression coefficient was taken as an estimate of the eye position sensitivity (*γ*).

In Purkinje cells with significant eye position sensitivity, the sensitivity to eye velocity (*β*') and the phase of eye velocity sensitivity (*ρ*') was recalculated with a correction for the eye position sensitivity as follows


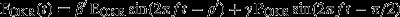
(3)

where F_OKR_ (t) is the firing rate response during OKR; E_OKR_ and P_OKR_ are the peak eye velocity and the peak eye position during the OKR, respectively, and *γ* is the sensitivity to eye position measured during spontaneous eye movements (see above).

To assess nonlinearities in the Purkinje cell responses, we first calculated average firing rate and eye velocity during each 10-ms bin of the OKR stimulus cycle. We then evaluated the linearity of the relationship between firing rate and eye velocity by comparing the slope of the relationship between firing rate and ipsiversive eye velocity with the slope of the relationship between firing rate and contraversive eye velocity (Lisberger et al. 1994).

## Results

### Responses of floccular Purkinje cells in mice during oculomotor behavior

To assess the signal content of Purkinje cells in the cerebellar flocculus of mice, we compared Purkinje cell responses during vestibularly and visually driven eye movements. We recorded eye movements and single unit activity of 110 Purkinje cells in 80 C57BL/6 adult (≥8 weeks old) mice (Fig.1). The mean firing rates during the VOR in the dark (VORD), VOR cancellation (VORC), and the optokinetic reflex (OKR) were 53.3 ± 2.8 sp/s, 52.1 ± 2.5 sp/s, and 55.3 ± 2.8 sp/s respectively (mean ± SEM, p > 0.60, one-way ANOVA). Cells were only included in the analysis if there was significant modulation of firing rate about the mean during at least one of these three test stimuli (p < 0.05, Rayleigh's test).

#### VOR in the dark

**Figure 2:**
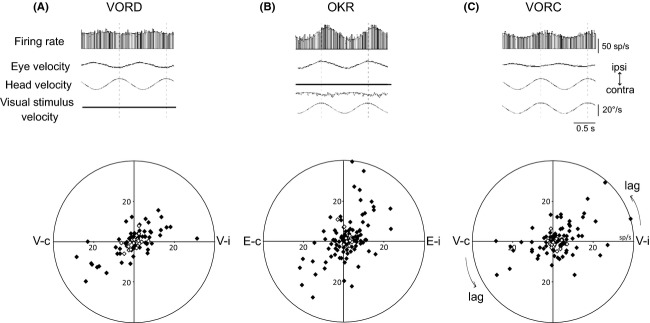
Purkinje cell responses during (A) performance of the VOR in the dark (VORD); (B) the optokinetic reflex (OKR); and (C) cancellation of the VOR (VORC). *Top*: Responses of a representative Purkinje cell, which responds to ipsiversive eye and head velocity. For illustration purposes, two identical cycles of the means are repeated. *Bottom*: Summary of the responses in a population of 110 Purkinje cells. Responses are plotted in polar coordinates; the distance from the origin represents the amplitude of the neural response (firing rate modulation relative to the mean) and the angle represents the phase of peak firing relative to the vestibular stimulus (for VORD and VORC) or eye movement (for OKR). A phase of 0° indicates peak firing during peak ipsiversive head velocity (V-i) or eye velocity (E-i). Counterclockwise rotation represents increased phase lag. Cells with a significant response to the test stimulus (p < 0.05, Rayleigh's test) are indicated by filled symbols; open symbols represent cells whose responses did not reach statistical significance.

The VOR was elicited by sinusoidal vestibular stimulation about an earth-vertical axis in total darkness (Fig.2A; 1 Hz, ±10°/s peak head velocity). The eye movement responses had an average gain of 0.35 ± 0.01, with the eye movements leading head movements by 27.0 ± 0.9°. Eighty-two of 110 Purkinje cells exhibited significant changes in firing rate during VORD (p < 0.05, Rayleigh's test; Fig.2A *bottom, filled symbols*). The distribution of the phases of the responses in different cells was not uniform; the phase tended to lag either peak ipsiversive or contraversive head velocity. Cells with peak firing during ipsiversive head movement outnumbered those with peak firing during contraversive head movement, 46 to 36 cells. In the first subpopulation, the phase of the mean neural response lagged peak ipsiversive head velocity by 24.8 ± 0.8° (n = 46); in the second subpopulation firing lagged peak contraversive head velocity by 24.0 ± 1.0° (n = 36).

#### Optokinetic reflex

To evaluate the encoding of nonvestibular signals by the Purkinje cells, their responses were recorded during performance of the OKR with the head stationary (Fig.2B). The OKR was elicited by a striped dome moving sinusoidally about an earth-vertical axis at 1 Hz with peak velocity of ±10°/s. The average gain of the OKR was 0.41 ± 0.01, with the phase of the eye movements lagging visual stimulus motion by 17.7 ± 0.6°. In the Purkinje cells, 101 of 110 cells responded with a significant change in firing rate during the OKR (p < 0.05, Rayleigh's test; Fig.2B *bottom, filled symbols*). Of those cells with a significant response during the OKR, the majority (65 of 101) increased their firing during ipsiversive eye velocity; in those cells, the phase of the mean neural response lagged peak ipsiversive eye velocity by 37.7 ± 1.1° (n = 65). In the 36 cells with peak firing during contraversive eye movement, firing lagged peak contraversive eye velocity by 24.5 ± 1.4°.

#### VOR cancellation

To isolate the contribution of vestibular versus eye velocity signals to a neuron's response, one approach has been to measure the cell's response as the animal cancels the eye movements driven by the VOR when tracking a visual stimulus that moves exactly with the head (VOR cancellation, VORC; Fig.2C). The eye movement gain during VORC (0.15 ± 0.01, phase −74.0 ± 1.4°) was substantially lower than during VORD (p < 0.01, t-test). Therefore, any contribution of eye movements to the Purkinje cell responses during VORD should be reduced during VORC, whereas the vestibular contribution should be the same, since the vestibular stimulus was the same. Ninety-two of 110 Purkinje cells exhibited significant firing rate modulation during VORC (p < 0.05, Rayleigh's test, Fig.2C *bottom, filled symbols*). Neurons with peak firing during ipsiversive head movement lagged peak head velocity by 17.6 ± 0.8° (n = 62), and neurons with increased firing during contraversive head movement lagged peak head velocity by 11.6 ± 1.3° (n = 30).

### Purkinje cell sensitivity to eye velocity and head velocity

Our recordings during VORD, VORC, and OKR indicate that individual floccular Purkinje cells in mice, like those in other species, carry both vestibular and nonvestibular signals. We calculated the sensitivity of each Purkinje cell to eye velocity and head velocity.

**Figure 3:**
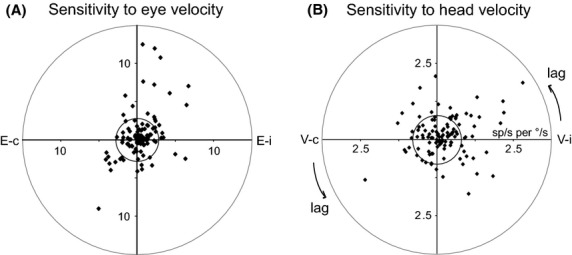
Sensitivity of Purkinje cells to eye and head velocity. Summary of the sensitivity of 110 Purkinje cells to eye velocity (A) and to head velocity (B), in polar coordinates. Distance from the origin represents the amplitude of eye or head velocity sensitivity, in spikes/s per °/s; the angle represents the phase of peak sensitivity, with a phase of 0° indicating peak firing during peak ipsiversive eye or head velocity. Counterclockwise rotation represents increased phase lag. Cells that responded to ipsiversive eye movements with an increase in firing rate were classified as Eye-ipsiversive (E-i; *right quadrants in* A) and cells that responded to ipsiversive head movements with an increase in firing rate were classified as vestibular-ipsiversive (V-i; *right quadrants in* B). Such responses are sometimes described as “type I.” Cells that responded to contraversive eye or head movement with an increase in firing rate were classified as E-c or V-c, or “type II.” Inner circles represent the mean sensitivity across the population. Note the different scales in panel A versus B.

Eye velocity sensitivity was calculated by dividing the amplitude of the firing rate response during the OKR by the peak eye velocity determined by Fourier analysis (Fig.3A). The mean sensitivity to eye velocity was 2.7 ± 0.2 sp/s per °/s for all Purkinje cells (n = 110), and 2.8 ± 0.3 sp/s per °/s for Purkinje cells that exhibited significant modulation of firing rate during the OKR (n = 101, p < 0.05, Rayleigh's test, Fig.2B).

Head velocity sensitivity was estimated by subtracting out the estimated contribution of eye velocity to a Purkinje cell's response during VORD, assuming a linear summation of signals encoding eye and head velocity (See Methods, eqn 2008). The contribution of eye velocity was calculated by scaling the measured eye velocity during VORD by the eye velocity sensitivity measured during OKR. This was subtracted from the Purkinje cell response during VORD, and the remainder was divided by peak head velocity to determine the vestibular sensitivity of the cell (Fig.3B). For all Purkinje cells, the average sensitivity to head velocity was 0.8 ± 0.1 sp/s per °/s (n = 110). Similar results were obtained if vestibular sensitivity was calculated by subtracting the eye velocity contribution to the Purkinje cell responses during VORC instead of VORD (Fig. S3) (0.9 ± 0.1 sp/s per °/s).

**Figure 4:**
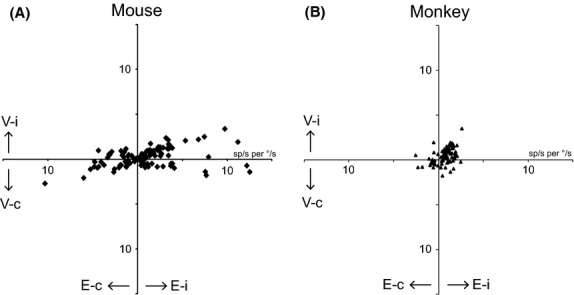
Subclassification of floccular Purkinje cells according to their sensitivity to eye velocity (abscissa) and head velocity (ordinate), for Purkinje cells in mice, recorded in this study (A), and Purkinje cells in monkey, adapted from Raymond and Lisberger (1997) (B). E-i: Eye-ipsiversive; E-c: Eye-contraversive; V-i: Vestibular-ipsiversive; V-c: Vestibular-contraversive.

Figure4A and Table 1 summarize the sensitivity of our sample of Purkinje cells to eye and head velocity. In Table 1, cells were categorized as having eye or head sensitivity if the calculated sensitivity was greater than 0.15 spikes/s per °/s (Stone and Lisberger 1990b). We also classified Purkinje cells using alternative criteria to account for the higher sensitivity of cells to eye velocity than to head velocity (Table S1A), to evaluate only those cells with relatively high sensitivity to eye or head velocity (Table S1B), to remove the influence of cells with the biggest responses (Table S1C), or to evaluate only those cells with relatively low sensitivity to eye and head velocity (Table S1D). The distribution of Purkinje cell responses was similar regardless of the criteria used. The great majority of cells carried both vestibular and nonvestibular signals. Moreover, cells were not equally distributed into the four main subclasses (p < 0.05 by Chi-square test). The largest single class of Purkinje cells increased their firing for ipsiversive eye and ipsiversive head velocity (E-i, V-i; Table 1 and Table S1; Fig.4A, *upper right quadrant*). The second largest class of Purkinje cells increased their firing for contraversive eye and contraversive head velocity (E-c, V-c; Table 1 and Table S1; Fig.4A, *lower left quadrant*).

**Table 1:** Subclassification of floccular Purkinje cells in mice

	E-c	E-i	No Eye	Total
V-i	13	49	0	62
V-c	22	19	0	41
No Head	2	4	1	7
Total	37	72	1	110

Subclassification of floccular Purkinje cells in mice. The criterion for inclusion of a cell in the E-i or E-c categories was a sensitivity to eye velocity ≥0.15 sp/s per °/s. The criterion for inclusion of a cell in the V-i or V-c categories was a sensitivity to head velocity ≥0.15 sp/s per °/s.

### Purkinje cell sensitivity to eye position

**Figure 5:**
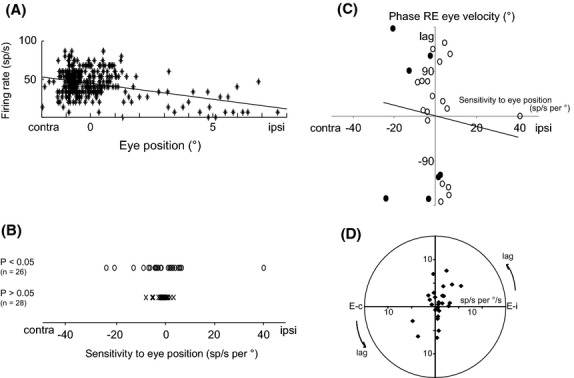
Sensitivity of Purkinje cells to eye position. (A) Example of a Purkinje cell with significant correlation between firing rate and eye position. Measurements were made during the spontaneous eye movements made by the mice while restrained with the head stationary in total darkness. Each point represents the average firing rates and eye position measured during a single, 150-ms period. Positive values on the *x*-axis indicate eye position ipsiversive to the recording site. The line indicates the linear regression between firing rate and eye position. (B) P-values and slopes of the linear regression between firing rate and eye position for each Purkinje cell tested. Twenty-six of fifty-four Purkinje cells showed significant correlation between firing rate and eye position. (C) The phase of firing relative to ipsiversive peak eye velocity during the OKR (*ordinate*), plotted as a function of the sensitivity to eye position (*abscissa*), in the 26 Purkinje cells with significant eye position sensitivity (*ovals in panel* B). Open and filled *ovals* indicate Purkinje cells categorized as E-i and E-c in Figure3A respectively. There was no significant correlation between the phase of the peak firing and the sensitivity to eye position (R = 0.012, p > 0.50). (D) Sensitivity to eye velocity, calculated with a correction for sensitivity to eye position, in the 26 Purkinje cells with significant eye position sensitivity (compare with Fig.3A). Results are plotted in polar coordinates, with distance from the origin representing the amplitude of eye velocity sensitivity, in spikes/s per °/s, and the angle representing the phase of peak sensitivity, with a phase of 0° indicating peak firing during peak ipsiversive eye velocity.

During the OKR, the phase of the peak firing of Purkinje cells lagged peak ipsiversive or contraversive eye velocity (Fig.2B). This phase lag could reflect a delayed response of Purkinje cells to eye velocity, a sensitivity to eye position, or both. To assess eye position sensitivity, Purkinje cells were recorded in the absence of vestibular or visual stimuli, and the dependence of firing on eye position was assessed. When animals sat with their head stationary in the dark, they made spontaneous saccadic eye movements, with eye position excursions of up to ∼10°. In roughly half of the Purkinje cells (26 of 54), there was a significant correlation between firing rate and eye position (Fig.5A and B). In 12 of these 26 cells, firing rate increased for more ipsiversive eye positions, with a mean sensitivity to eye position of 7.3 ± 3.1 sp/s/°. In the remaining 14 cells, firing rate increased for more contraversive eye positions, with a mean sensitivity to eye position of 7.1 ± 1.9 sp/s/° (n = 14).

Sensitivity to eye position did not account for the phase lag in the firing of the Purkinje cells during the OKR. There was no significant correlation between the eye position sensitivity of a Purkinje cell and the phase of its firing during the OKR (R = 0.012; p > 0.50, n = 26), even in the 26 Purkinje cells with significant sensitivity to eye position (Fig.5C). Moreover, when the sensitivity of these 26 cells to eye velocity was recalculated with a correction for their eye position sensitivity, the correction had little impact on the results (Fig5D). To estimate the contribution of eye position to the simple spike response of each Purkinje cell during the OKR, the eye position excursion during the OKR (±0.65° on average) was multiplied by the eye position sensitivity of the cell. This estimated contribution of eye position was subtracted from the Purkinje cell's response during the OKR, and the remaining response was used to recalculate the cell's sensitivity to eye velocity. With this correction for eye position, the eye velocity sensitivity of the 26 Purkinje cells was 3.5 ± 0.5 sp/s per °/s (Fig.5D), compared to 2.9 ± 0.5 sp/s per °/s in these same cells without the correction for eye position. For the E-i cells, the correction for eye position produced a moderate reduction in the mean phase lag of the cells' responses relative to eye velocity from 34.1° to 22.0°. In the small subset of E-c cells with significant eye position sensitivity (n = 8), correction for eye position completely eliminated the lag of their response relative to eye velocity (from a mean 10.8° lag to a lead of 30.3°).

### Nonlinearities in the encoding of eye velocity

**Figure 6:**
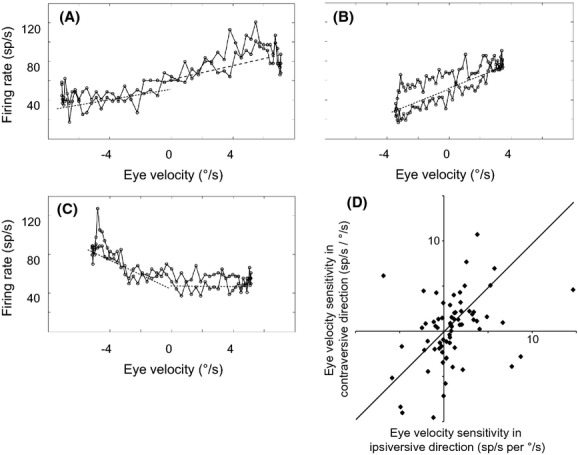
Linear and nonlinear encoding of eye velocity. (A–C) Relationships between firing rate and eye velocity in three different Purkinje cells. Each point represents the average firing rate and eye velocity during 1/100 of the stimulus cycle of the OKR. The cells in panels B and C illustrate two types of nonlinearities that were observed (see text for details). (D) Comparison of the sensitivity to eye velocity toward (ipsiversive, *abscissa*) and away from (contraversive, *ordinate*) the side of recording. Sensitivity was calculated from plots like those in (A–C), with each point representing the slopes of the regression lines for ipsiversive and contraversive eye velocity. Positive values of sensitivity indicate that firing rate increased for ipsiversive or decreased for contraversive eye velocity, and negative values indicate that firing rate decreased for ipsiversive or increased for contraversive eye velocity. The diagonal line has a slope of 1.

Some of the Purkinje cells in the floccular complex of mice exhibited nonlinearities in the relationship between firing rate and eye velocity, similar to those previously reported in monkeys (Lisberger et al. 1994). Such nonlinearities can be observed in plots of average firing rate versus eye velocity at each phase of the OKR stimulus cycle (Fig.6). For some cells, the relationship between firing rate and eye velocity was close to linear (Fig.6A), but in others, nonlinearities could be detected. In some cells, there was “hysteresis”, such that firing rate was different for a given eye velocity, depending on whether the eye was accelerating or decelerating (Fig.6B), as one would expect from a phase lag in the neural response to the sinusoidal eye velocity profile (Fig.2B). In addition, some cells had a different sensitivity to eye velocity in the ipsiversive and contraversive directions (different slopes for positive vs. negative values of eye velocity, Fig.6C; Fig6D summary plot). Such asymmetries could not be explained by cutoff at or near zero firing rate during the OKR in the “off” direction for the cell, because in all cells, the modulation of firing rate was less than the mean firing rate (Fig. S4).

### Summation of vestibular and eye-movement related signals in a Purkinje cell

**Figure 7:**
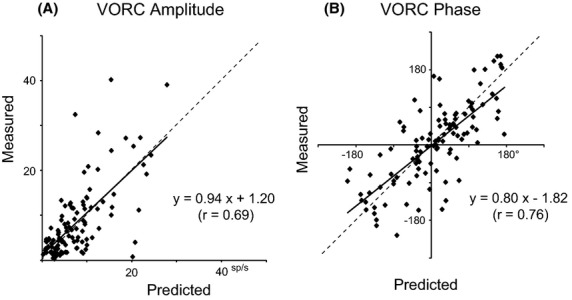
Comparison of the amplitude (A) and phase (B) of Purkinje cell responses measured during VORC (*ordinate*) with those predicted from the responses measured during the OKR and VORD (*abscissa)*. Points falling on the dashed line of slope 1 would indicate perfect prediction.

Despite the nonlinearities described above, Purkinje cell responses in the floccular complex of monkeys and goldfish can be well-approximated by a linear summation of the responses to the head velocity and eye velocity inputs (Lisberger and Fuchs 1978a; Miles and Eighmy 1980; Pastor et al. 1997; Hirata and Highstein 2000). We evaluated this in mice by testing the extent to which the response of each Purkinje cell during VORC could be predicted from the responses during VORD and OKR. The response during VORC was predicted from the linear combination of the eye and head velocity during VORC, weighted by the eye and head velocity sensitivity extracted from the responses during VORD and OKR. There was a significant correlation between the amplitude of the actual and predicted responses (p < 0.01, R = 0.69, slope = 0.94, Fig.7A). In addition, there was a significant correlation between the actual and predicted phase of the neural responses (p < 0.01, R = 0.76, slope = 0.80, Fig.7B). Thus, to a first approximation, eye and head velocity signals seem to be linearly combined to drive firing in the floccular Purkinje cells in mice, over the range of head and eye velocities used in this study.

## Discussion

We assessed the signal content of floccular Purkinje cells in awake mice performing eye movement responses to both vestibular and visual stimuli. In a population of 110 Purkinje cells responding to one or more of the test stimuli, the responses had several general characteristics consistent with what has been reported previously in other species. First, individual Purkinje cells in the flocculus carried both vestibular and nonvestibular signals. Second, the number of Purkinje cells that responded to ipsiversive eye movement or ipsiversive head movement outnumbered those that responded to contraversive eye or head movement. Third, the largest single category of Purkinje cells was those that responded to both ipsiversive eye and head velocity (E-i, V-i). Thus, the signal content of Purkinje cells in the flocculus of mice was qualitatively similar to that in other species. Quantitatively, however, there was one notable difference between mice and other species; Purkinje cells in mice were substantially more sensitive to eye velocity than head velocity.

### Nonvestibular signals carried by the floccular Purkinje cells

In our sample, 92% of the Purkinje cells (101 out of 110) carried nonvestibular signals, as evidenced by their responses during visually driven eye movements with the head stationary (OKR). Of these, 64% had peak firing during ipsiversive eye velocity (E-i). This is similar to the percentage of eye-movement sensitive neurons that are E-i in the floccular complex of goldfish (65%) (Pastor et al. 1997), monkeys (75–80%) (Lisberger and Fuchs 1978a; Markert et al. 1988), and rabbits (80%) (Nagao 1989).

The average eye velocity sensitivity in our sample of Purkinje cells in mice was 2.8 sp/s per °/s. This is higher than the average eye velocity sensitivity of Purkinje cells reported in other species: 0.90–1.02 sp/s per °/s in monkeys (Lisberger and Fuchs 1978a; Miles and Eighmy 1980; Stone and Lisberger 1990b), 0.70–0.98 sp/s per °/s in goldfish (Pastor et al. 1997), and 1.62–1.74 sp/s per °/s in rabbit (Miyashita 1984; Nagao 1991).

Notably, the average eye velocity sensitivity we measured, although higher than reported in other species, was no higher than that reported in two previous studies in mice, which used a video method rather than the eye coil method to measure eye movements (Goossens et al. 2004; Yoshida et al. 2007). Some investigators have raised the concern that an eye coil could load or otherwise impede eye movements in mice and thereby influence Purkinje cell firing via efference copy or proprioceptive inputs (Stahl et al. 2000; Katoh et al. 2007). If so, then one would expect to record higher eye velocity sensitivity in neurons in the oculomotor pathway (stronger motor command required to achieve the same eye velocity) and altered dynamics using the eye coil versus video system. However, the average eye velocity sensitivity we measured using the eye coil system was similar to (3–3.5 sp/s per °/s, Yoshida et al. 2007) or lower than the values reported previously in mice (6.5 sp/s per °/s, Goossens et al. 2004). Likewise, the phase lags in our Purkinje cell sample during the OKR were similar to those in the previous studies that used video. Therefore, it is unlikely that the method for measuring eye movements significantly influenced the results. Rather, the higher sensitivity to eye velocity measured in mice likely reflects a true functional difference between species.

The higher sensitivity of mouse Purkinje cells to eye velocity could enable them to use more of the dynamic range of their firing rates to represent the range of possible eye velocities, since peak velocities reported for visually driven eye movements in mice are generally lower than those in monkeys: 3–7.8°/s in mice (Iwashita et al. 2001; Faulstich et al. 2004) versus 40–140°/s in monkeys (Barmack 1970; Fuchs et al. 1994). In addition, the lower OKR gain in mice compared with monkeys or goldfish results in higher image speeds on the retina, and these visual signals may contribute to the responses of the Purkinje cells during the OKR.

The phase of peak firing in the Purkinje cells lagged peak eye velocity by about 20–40° during the OKR at 1 Hz (Fig.3) or 0.8 Hz (Goossens et al. 2004; Yoshida et al. 2007). Approximately half of the cells had sensitivity to eye position that could contribute to this phase lag, however, for the large majority of cells, the eye position sensitivity could not fully account for the phase lag of the cells' firing relative to eye velocity (Fig.5). Thus, a delayed response to eye velocity may also contribute to the phase lag. This may explain why Purkinje cell responses at different OKR frequencies could not be fit using a single set of parameters for eye velocity and eye position sensitivity (Goossens et al. 2004).

### Vestibular signals carried by the floccular Purkinje cells

Because the VOR behavioral paradigm is used to analyze the molecular-genetic mechanisms of cerebellum-dependent learning in mice, the main goal of the current study was to assess the signal content of Purkinje cell responses in awake mice during performance of the VOR. We estimated the vestibular contribution to each Purkinje cell's firing by subtracting the estimated contribution of eye velocity from the total response during VORD, and found that 63% of Purkinje cells increased their firing in response to ipsiversive head movement. This percentage of vestibular-ipsiversive (V-i) cells in mice is similar to that in rats (60%; Blanks and Precht 1983) and monkeys (72%; Lisberger and Fuchs 1978a; Stone and Lisberger 1990a) but smaller compared to goldfish (100%; Pastor et al. 1997). The vestibular sensitivity in mice (0.8 sp/s per °/s) was similar to that reported in monkey (0.68 sp/s per °/s, Lisberger and Fuchs 1978a; 1.03 sp/s per °/s, Miles et al. 1980) and in goldfish (1.04 sp/s per °/s, Pastor et al. 1997).

### Subclassification of floccular Purkinje cells

Individual floccular Purkinje cells in mice could carry both vestibular and nonvestibular signals, consistent with a role of these cells in coordinating the eye movement response to visual and vestibular inputs. Indeed, most of the Purkinje cells carried signals related to both head and eye velocity (Table 1). Moreover, despite nonlinearities observed in some cells (Fig.6), Purkinje cell responses could be estimated, to a first approximation, as a linear combination of vestibular and eye velocity inputs (Fig.7), as reported previously in monkeys and goldfish (Stone and Lisberger 1990b; Pastor et al. 1997; Hirata and Highstein 2000).

The breakdown of mouse floccular Purkinje cells into specific subclasses by vestibular and eye movement response type (Table 1) is similar to that in other species (Stone and Lisberger 1990a; Pastor et al. 1997). A majority of Purkinje cells were E-i, V-i or E-c, V-c. In monkeys and goldfish, such cells have been called Horizontal Gaze Velocity Purkinje cells (HGVPs) because their sensitivity to eye velocity in the head and to head velocity in the world in the same direction makes their responses closely related to eye velocity in the world, or gaze velocity (Fig.4B, *upper right quadrant*). In these neurons, the vestibular and efference copy signals would tend to cancel during VORD, when the head and eyes move in opposite directions. Accordingly, the responses of the Purkinje cells in our sample were, on average, smaller during VORD (6.9 ± 0.7 sp/s) than during OKR (10.3 ± 0.9 sp/s) or VORC (8.4 ± 0.8 sp/s). In other species, the sensitivity of Purkinje cells to eye velocity and head velocity is similar (see, for example, Fig.4B), so that the two inputs tend to balance during performance of the VOR with a gain of ∼1.0 (Stone and Lisberger 1990a). In mice, the eye velocity sensitivity was, on average, 3.5 times higher than the sensitivity to head velocity (Fig.4A), so that the two inputs would balance with an eye movement gain of ∼0.29 (1/3.5), which is close to the measured VOR gain of 0.35 ± 0.01. Thus, within each species, the sensitivity of the floccular neurons to head and eye velocity may be tuned to the behavioral parameters.

### Signal content of neurons in the flocculus versus downstream in the vestibular nuclei

The flocculus gets input from neurons in the vestibular nuclei and sends output signals to the vestibular nuclei (Balaban et al. 1981; Zhang et al. 1993; Lisberger et al. 1994), however, there appear to be differences in the signal content of cells in these two brain regions. Neurons in the medial vestibular nuclei (MVN) of alert C57BL/6 mice (Beraneck and Cullen 2007) and paralyzed mice (Grusser-Cornehls et al. 1995b; Baurle et al. 1997) have a sensitivity to head velocity of approximately 0.4–0.6 sp/s per °/s, similar to Purkinje cells in the flocculus. However, Beraneck and Cullen (2007) reported eye position signals, but little or no eye velocity sensitivity of cells in the MVN of mice, in contrast with our estimation of high eye velocity sensitivity of Purkinje cells. This previous study of MVN used constant velocity optokinetic stimuli, rather than sinusoidal stimuli, and this methodological difference could contribute to the apparent difference between the Purkinje cells and MVN neurons, however, it also could reflect a true difference in signal content. Thus, additional studies are necessary to determine whether and how the large eye velocity signals carried by the Purkinje cells influence downstream structures such as the MVN.

## Supporting Information

**Figure S1.** Sample of the recording sites in the flocculus.

**Figure S2.** Example raw traces of eye movements.

**Figure S3.** Sensitivity of Purkinje cells to head velocity calculated by using Purkinje cells' response during OKR and VORC.

**Figure S4**. Amplitude of the modulation of Purkinje cell activity during (A) VORD, (B) OKR, and (C) VORC as a function of the average firing rate of Purkinje cells.

**Table S1.** Subclassification of floccular Purkinje cells in mice using different criteria.
